# Medusa: A tool for exploring and clustering biological networks

**DOI:** 10.1186/1756-0500-4-384

**Published:** 2011-10-06

**Authors:** Georgios A Pavlopoulos, Sean D Hooper, Alejandro Sifrim, Reinhard Schneider, Jan Aerts

**Affiliations:** 1 Katholieke Universiteit Leuven, Faculty of Engineering - ESAT/SCD, Kasteelpark Arenberg 10, 3001 Leuven-Heverlee, Belgium; 2 European Molecular Biology Laboratory (EMBL), Structural and Computational Biology, Meyerhofstrasse 1, 69117, Heidelberg, Germany; 3 Department of Genetics and Pathology, Uppsala University, SE-751 85 Uppsala, Sweden; 4 Luxembourg Centre for Systems Biomedicine (LCSB), University of Luxembourg, Campus Limpertsberg, 162A, Avenue de la Faïencerie, L-1511, Luxembourg

**Keywords:** graph, visualization, biological networks, clustering analysis, data integration

## Abstract

**Background:**

Biological processes such as metabolic pathways, gene regulation or protein-protein interactions are often represented as graphs in systems biology. The understanding of such networks, their analysis, and their visualization are today important challenges in life sciences. While a great variety of visualization tools that try to address most of these challenges already exists, only few of them succeed to bridge the gap between visualization and network analysis.

**Findings:**

Medusa is a powerful tool for visualization and clustering analysis of large-scale biological networks. It is highly interactive and it supports weighted and unweighted multi-edged directed and undirected graphs. It combines a variety of layouts and clustering methods for comprehensive views and advanced data analysis. Its main purpose is to integrate visualization and analysis of heterogeneous data from different sources into a single network.

**Conclusions:**

Medusa provides a concise visual tool, which is helpful for network analysis and interpretation. Medusa is offered both as a standalone application and as an applet written in Java. It can be found at: https://sites.google.com/site/medusa3visualization.

## Background

The analysis and interpretation of complex relationships between biological molecules, networks and concepts presents a major bottleneck in systems biology. Different types of networks such as protein-protein interaction (PPI) networks, biochemical networks, transcriptional regulation networks, signal transduction or metabolic networks are significantly different in structure but often share characteristics and properties that need to be further explored in detail. Understanding the complexity of such systems, which often contain thousands of nodes and thousands of connections, is neither an easy nor trivial task. Therefore, there is an increasing need for advanced, efficient and informative visualization tools. In the field of data integration, the analysis of heterogeneous data from different data sources can be very complicated. In addition, the simultaneous analysis of heterogeneous networks within the same view increases the complexity even more and therefore the analysis of such graphs is becoming incomprehensible. While many different approaches from graph theory, as reviewed in [1], try to reveal patterns, characteristics, properties and information well hidden in different types of networks, the implementation of such algorithms presents a major bottleneck, especially for researchers who are not computationally experienced.

Currently, many visualization tools [2] try to cope with the increasing complexity of network analysis. Already established tools include Cytoscape [3], Cytoscape Web [4], Osprey [5], Ondex [6], Medusa [7], Arena3D [8], Pajek [9], BioLayout Express^3D ^[10] and others [2]. While most of these tools try to efficiently visualize complex networks using informative views, they often lack basic statistics that can help to interpret the visualization or clustering algorithms to directly analyze a network. Cytoscape, which is currently a golden standard visualization tool in the areas of network analysis and visualization, currently tries to cope with these issues by using plugins. Its main strength is its architecture that allows plugins mainly developed by experienced users. Currently Cytoscape comes with a broad variety of plugins with diverse functionality, 56 of those are used for analyzing existing networks (e.g ClusterMaker [11]) and 9 of them aim to functionally annotate and enrich the network (i.e BiNGO [12]). A list of plugins can be found in: http://chianti.ucsd.edu/cyto_web/plugins/.

Under the guidance of targeted end-users, we developed Medusa, which is specifically designed to address tasks from the areas of network visualization, data analysis and data integration. It currently hosts a variety of layout and clustering algorithms to directly analyze the networks and reveal hidden patterns. Its new GUI makes Medusa user friendly and easier to use comparing to its previous version. It is an open source project, which gives access to the code for programmers that want to directly modify and adjust it to the needs of various projects.

The Medusa tool was first released in 2005 [7]. In this paper, we present a significant update, based on a complete redesign of the underlying infrastructure and implementation of a large number of requested features.

## Implementation

### Features

In this section we present major changes/updates to Medusa, providing significant additional functionality. This version of Medusa comes with a friendlier interface and is offered both as an applet and a standalone application. It is enriched with several layout and clustering algorithms to provide intuitive views and high quality analysis while it is becoming more interactive to cope with the high complexity of the networks. A summarized description of the features of Medusa 3.0 application is shown in Table 1 while advantages comparing to previous versions are shown in Table 2.

**Table 1:** Features of Medusa

Layout algorithms	Clustering Algorithms	Features
Grid	Predefined clustering	Multi-edged connections

Random	k-Means	Curves, Lines, Arrows

Circular	Spectral	Interactivity

Hierarchical	Affinity Propagation	Compatibility with other tools

Force-Directed		Offered as an applet

Spring Embedding		Collapse/Expand node

Distance geometry		Search functionality

Parallel Coordinates		Color schemes

		Isolation of subset of edges

**Table 2:** Medusa vs previous versions

Characteristics	Previous versions	Medusa 3.0
Curves, Lines, Arrows	X	X

Collapse/Expand node	X	X

Multi-edged connections	X	X

Save/Reload the status of the network	X	X

Load background static images	X	X

Circular layout		X

Hierarchical layout		X

Force-Directed	X	X

Spring Embedding	X	X

Distance geometry layout		X

Parallel Axes		X

Clustering Algorithms		X

Predefined clustering		X

Save to other formats		X

New GUI		X

Isolation of edges when dragging nodes		X

Richer Color schemes		X

Richer search functionality		X

Applet with higher parameterization		X

Applet with richer functionality		X

Simple network statistics		X

Open source	X	X

#### Main View

**Figure 1:**
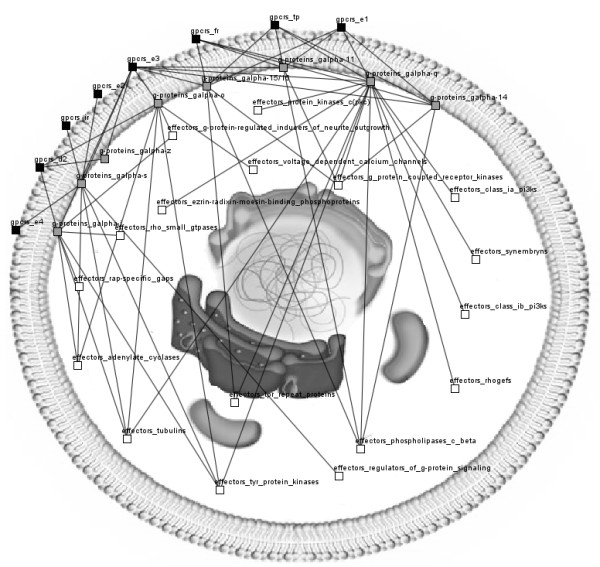
**Visualization of human postanoid receptors and their interactions**. GPCR transmembrane proteins (black) are classified in subfamilies whereas G-proteins (grey) and effectors (white) are classified in families according to the Human-gpDB database [23]. A preloaded image of a cell shows the signal transduction from the outer to the inner part of the cell.

In Medusa, we use a 2D panel to project networks consisting of nodes with their connections. For more conceptual and descriptive visualizations, users can load an external image as a background. An example is shown in Figure 1 where signal transduction paths are overlaid on top of an image of a cell showing how signals are transferred from the outer to the inner part of the cell. The nodes are manually placed in such a way that the representation is self-explanatory.

#### Nodes

As Medusa is specifically designed to integrate heterogeneous data from different data sources under the same network, users can enrich the networks by defining node parameters such as annotation strings, URL addresses, shapes, coordinates or colors. This way, users can visually navigate through similar or different types of nodes.

#### Edges

Medusa currently supports visualization for both directed and undirected graphs. In the case of weighted graphs, confidence or similarity scores can be shown, by adjusting the color intensity of the line. For multi-edged networks, Medusa utilizes Bezier curves to support up to 8 different types of connections between two bioentities. Each type of connection is characterized by a unique color. This feature is very powerful when one wants to display information originating from various data sources. Two genes for example may co-occur in literature, co-express in one experiment or be evolutionary related (3 different types of connections).

A major problem that occurs when the number of edges increases is that it becomes very difficult for a user to follow which nodes are interconnected with each other. To overcome this problem in such cases of dense networks, Medusa gives the opportunity to the users to isolate the connections of specific nodes by dragging them. This way, all of the connections of the network are instantly hidden while only the connections of the selected nodes of interest are visible, thus providing a much clearer view. Finally, The number of connections can get filtered down according to user-defined thresholds.

#### Interactivity

The tool is highly interactive and easy to use. Users can drag nodes and place them anywhere, add new ones on the fly or delete them. Groups of nodes can furthermore be merged into one single node or expanded. Standard operations such as selection of sub-networks, zooming in/out, rotation, scaling and translation are also supported. Medusa comes in addition with embedded text search functionality. This way, nodes can be searched by name or by annotation and sets of nodes can be selected either graphically or by using text regular expressions.

#### Exporting

While Medusa currently supports its own simple file format as described in https://sites.google.com/site/medusa3visualization/file-format, to complement its functionality with other already established tools, networks can now be saved in Pajek [9], Cytoscape [3], BioLayout Express^3D ^[10], Arena3D [13] and GraphViz library formats. The status of the network can be saved and reloaded anytime and graphs can be exported as image files.

#### Implementation

Medusa is offered both as a java standalone application and as an applet. The java applet comes with limited functionality compared to the standalone application though. Launching Medusa as an applet makes the application highly versatile in contrast to the current visualization tools that are either used locally as standalone applications or produce static images to be integrated in web pages. The Medusa web application is highly portable, easy to use, and highly interactive while it can get embedded in any project that requires network visualization though the web. In the current version, the applet comes with much richer functionality comparing to the previous versions. Now users, can hide or show connections of interest on the fly, apply any layout or clustering algorithm that is introduced in the standalone application or enrich the network by adding annotations, labels or URL addresses for each node (double click to redirect).

### Layouts

Several node layout algorithms are implemented to result in clearer network representations. Simpler layouts distribute the nodes randomly, on a grid or on a circle. The Fruchterman-Reingold [14] force directed layout algorithm tries to minimize the crossovers between the lines. A second algorithm based on distance geometry [15] places the nodes in such a way that the more correlated two nodes are, the closer they are placed to each other. A third hierarchical layout algorithm places the nodes in a hierarchy (tree-like structure). Such a layout is useful for example to visualize Gene Ontology [16] graphs. Inspired by the concept of Arena3D where nodes are separated onto different layers, Medusa tries to present a similar type of visualization with the use of parallel coordinate axes. Besides defining the coordinates of the nodes automatically by using any of the aforementioned layout algorithms, users are able to manually define the coordinates of the nodes in the input file. Therefore, external layout algorithms can be used to pre-calculate the coordinates of the nodes.

### Clustering

Clustering approaches implement algorithms and methodologies that tend to group elements together according to similar features or characteristics. Medusa currently supports a set of clustering algorithms such as the Affinity Propagation [17], k-Means [18] and spectral clustering [19]. To represent the clusters, we place nodes on circles and assign a unique color to each respective cluster, enabling visual analysis of the clustering results. The results can also be exported as text files to make external analysis possible. Medusa currently supports the visualization of pre-calculated clustering data performed by external applications in case users want to use their own algorithms to cluster data.

## Results

Medusa is already widely used in several diverse case studies that need the support of network visualization. In this section we show the spectrum of the biological questions that Medusa can answer through its input to various existing projects.

### Clustering

Medusa was used to identify and extract protein complexes from a protein-protein interaction yeast dataset [20] as presented in [21]. In a recent study, we benchmarked various clustering algorithms using the jClust clustering package [22]. While in the aforementioned study, jClust was used as an external application to cluster data and Medusa as a front-end application to visualize the results, now Medusa is able to reproduce such results easily since most of the clustering algorithms are now offered within the Medusa application.

### Signal Transduction

Taking advantage of its layout and color schemes, Medusa was used to visualize signal transduction from the outer to the inner part of the cell. This is demonstrated in Figure 1, which uses the image of a cell as background while nodes are manually placed in a clear way to visualize data from the Human-gpDB database [23]. This database holds information about G-Proteins and their interactions with human GPCRs and effectors and on how various stimuli activate GPCRs transmembrane proteins. The effectiveness of this visualization has led the Human-gpDB project to deliver the results visually within the browser.

### Data Integration

One of the strong characteristics of Medusa is its ability to support multi-edged connections between two nodes. Two nodes can be connected with more than one way, each line representing a different type of connection or a different concept. For example two genes might be evolutionary related or co-occur in literature or co-express in a set of experiments (3 types of connections between the genes). This way, heterogeneous data coming from various data sources can now get efficiently interconnected and visualized. Medusa. Medusa serves as an excellent front-end application to support visualization of the STRING [24] database which holds information about interactions between different data types that come from various sources (e.g. gene fusion, co-occurrence, experiments, databases, text mining, homology etc.). Medusa is not a tool to integrate data but a tool to visualize already integrated data from various data sources such as STRING database.

Medusa was also used as a front-end for the COAL application [25] to integrate phenotypic metadata and protein similarity in Archaea using a spectral bi-partitioning approach. For each of the bioentities, Medusa provides links to a functional summary, a characteristic member sequence and adjacent links to parent clusters and sub-clusters, wherever available.

Having demonstrated the broad spectrum of problems that this new version of Medusa is able to address, we believe that Medusa can serve as a great front or back end application for different case studies to analyze and visualize biological networks.

## Discussion

We present Medusa as an alternative and complementary tool to other software packages such as Cytoscape [3], Pajek [9], Arena3D [13] and Ondex [6]. Given the strengths of the aforementioned visualization tools, Medusa provides several advantages compared to the other packages and occupies its own niche. For example, although Pajek [9] is richer in functionality, it requires complicated input files and cannot be used within a browser. Arena3D [13] is mainly aimed at displaying multilayered graphs and can be computationally expensive for larger datasets. Ondex [6] is an application implemented to retrieve data from databases and cannot be used in simpler cases. Cytoscape [3] finally, is one of the leading applications in the field. It provides a plugin framework for loading additional functionality, mostly focusing on enriching and annotating the networks. Similarly to Cytoscape, Medusa is released under an open source license with the advantage that is easier to modify and adjust to the needs of individual projects. It is noticeable that Cytoscape is supported by a whole consortium and is richer in functionality but also more complex to understand and modify the code. Combined with its clear implementation, Medusa allows the end-users to easily change any functionality from the GUI to the core itself.

Most visualization tools in this area lack integration with web technologies. Cytoscape recently released Cytoscape web [4] to address this issue. To our knowledge, however, this is the only application so far because most of the other available tools produce static images to be embedded in web applications. On the other hand, Medusa provides functionality to easily incorporate network visualizations within web applications. Compared to Cytoscape web, we have also found that Medusa performs faster within the browser for larger networks.

Future work will involve automated integration of Medusa with various data sources through web services in addition to compatibility with established file formats such as SBML [26] and PSI-MI [27]. Implementation of ranking algorithms using certain attributes of the network is also planned. Sorting the nodes according to characteristics such as connectivity, degree, matching index, closeness, betweenness and eigenvector centrality or clustering coefficient will highlight the statistically and functionally more significant nodes of the network. Most of the aforementioned rankings are well documented in [1]. We finally aim to use the Processing rendering machine [28] to replace the current graphics in order increase the quality of the graphics and to expand the interactivity.

Medusa is already widely used and comes as a very easy to use tool, able to represent information within web browsers in a very simplified way. Medusa's combination of visualization, analysis, user friendliness, open access, and browser compatibility provide researchers a fast and easy way to visualize and analyze their data.

## Conclusions

Medusa is a powerful tool that combines concepts from both the areas of network analysis and network visualization. It now comes with embedded layout and clustering algorithms while the new GUI is user friendlier and easier to use. It is offered both as a standalone application and an applet thus it is very easy to integrate with web applications to present data in a web browser. We believe that Medusa can be applied in various interdisciplinary fields and help researchers to present, analyze or explore data in an easy and self-explanatory way.

## Availability and Requirements

**Documentation**: https://sites.google.com/site/medusa3visualization

**Download**: http://sourceforge.net/projects/graph-medusa/files

**JavaDoc**: https://sites.google.com/site/medusa3visualization/download

**User feedback**: http://medusa.userecho.com

**Operating System**: Platform Independent

**Programming Language**: Java

**Requirements**: JRE 1.6 or higher

**Operating System**: Platform Independent

**Source Code**: Open source, Free for academic use

**License**: GNU General Public License (GPL)

## Competing interests

The authors declare that they have no competing interests.

## Authors' contributions

GAP implemented the clustering, the layout algorithms and updated the interface. SDH initiated the project and wrote the core code of the application. AS, RS and JA gave crucial suggestions on how to enrich the functionality of Medusa. RS and JA supervised the project. All of the authors contributed to the manuscript. All of the authors have read and approved the manuscript.
